# Large-scale ecological networks do work in an ecologically complex biodiversity hotspot

**DOI:** 10.1007/s13280-015-0697-x

**Published:** 2015-09-16

**Authors:** Michael J. Samways, James S. Pryke

**Affiliations:** Department of Conservation Ecology and Entomology, Stellenbosch University, Private Bag X1, Matieland, 7602 South Africa

**Keywords:** Conservation corridors, Functional connectivity, Biodiversity, Mitigation, Retrospective analysis

## Abstract

Landscape-scale ecological networks (ENs) are interconnected conservation corridors of high-quality habitat used to mitigate the adverse effects of landscape fragmentation and to connect with protected areas. The effectiveness of ENs for biodiversity conservation and ecosystem function has been challenged. Here we show how an extensive system of ENs of remnant historic land was put in place at a large spatial scale (>0.5 million ha) in a plantation forestry context in a global biodiversity hotspot in southern Africa. These ENs can maintain indigenous and historic compositional and functional biodiversity, even in an area prone to the challenging effects of El Niño. Furthermore, ENs increase the effective size of local protected areas. Socio-ecological solutions and financial viability are also integrated as part of practical implementation of ENs. By adopting a retrospective analytical approach, biodiversity is maintained while also having productive forestry, making this a powerful agro-ecological approach on a large conservation-significant scale.

## Introduction

When strips of remnant habitat (conservation corridors) are interwoven across the landscape to improve structural and functional connectivity in all directions, the configuration is an ecological network (EN) (Jongman 1995). ENs, structurally and functionally, aim to connect formally proclaimed protected areas (PAs) and other areas of high natural value across transformed landscapes, so as to mitigate the effects of fragmentation of remnant natural areas (Jongman 1995; Hepcan et al. 2007; Samways 2007; Gurrutxaga et al. 2010; Samways et al. 2010). Furthermore, ENs must continue to function adequately over time (Auffret et al. 2015). However, Boitani et al. (2007) maintain that there is still relatively little scientific evidence that ENs are effective for the long-term conservation of biodiversity or of ecosystem processes. We address this challenge here, showing that indeed they can be of major conservation significance, and that ENs as a conservation approach should be adopted more widely across the globe, especially as they address five of the Aichi Biodiversity Targets (4, 7, 11, 14, 19).

The opportunities for instigating and maintaining remnant ENs in the face of widespread landscape fragmentation is rapidly diminishing. This means that we must find efficient selection and justification procedures for implementing ENs while at the same time addressing Boitani et al.’s (2007) concerns.

In reality, and given the urgency of addressing the biodiversity crisis, there is not enough time to gather all the baseline data to provide an immediate, robust, and resilient solution (Lindenmayer et al. 2013). This means that an intuitive solution based on fundamental conservation knowledge has to be invoked (Meir et al. 2004). This solution may have great value for effective conservation, and it can then be tested through strategic scientific research. To fully appreciate this rapid-implementation approach, we need to move away from the traditional one of spending much valuable time gathering an exhaustive database required to define the problem in the first place. Rather, we need a new solution that goes to the heart of the challenge: conserving biodiversity and maintaining ecosystem processes, especially historic ones (Murcia et al. 2014), as fast as possible. This can be done by retrospective analysis or ‘thinking backwards’ (Grose 2014, 2015). With this alternative approach, the way forward is framed as an inverse problem, where the aim is an immediate solution, and then data are gathered in an efficient and direct way to find the solution (Grose 2014).

**Fig. 1:**
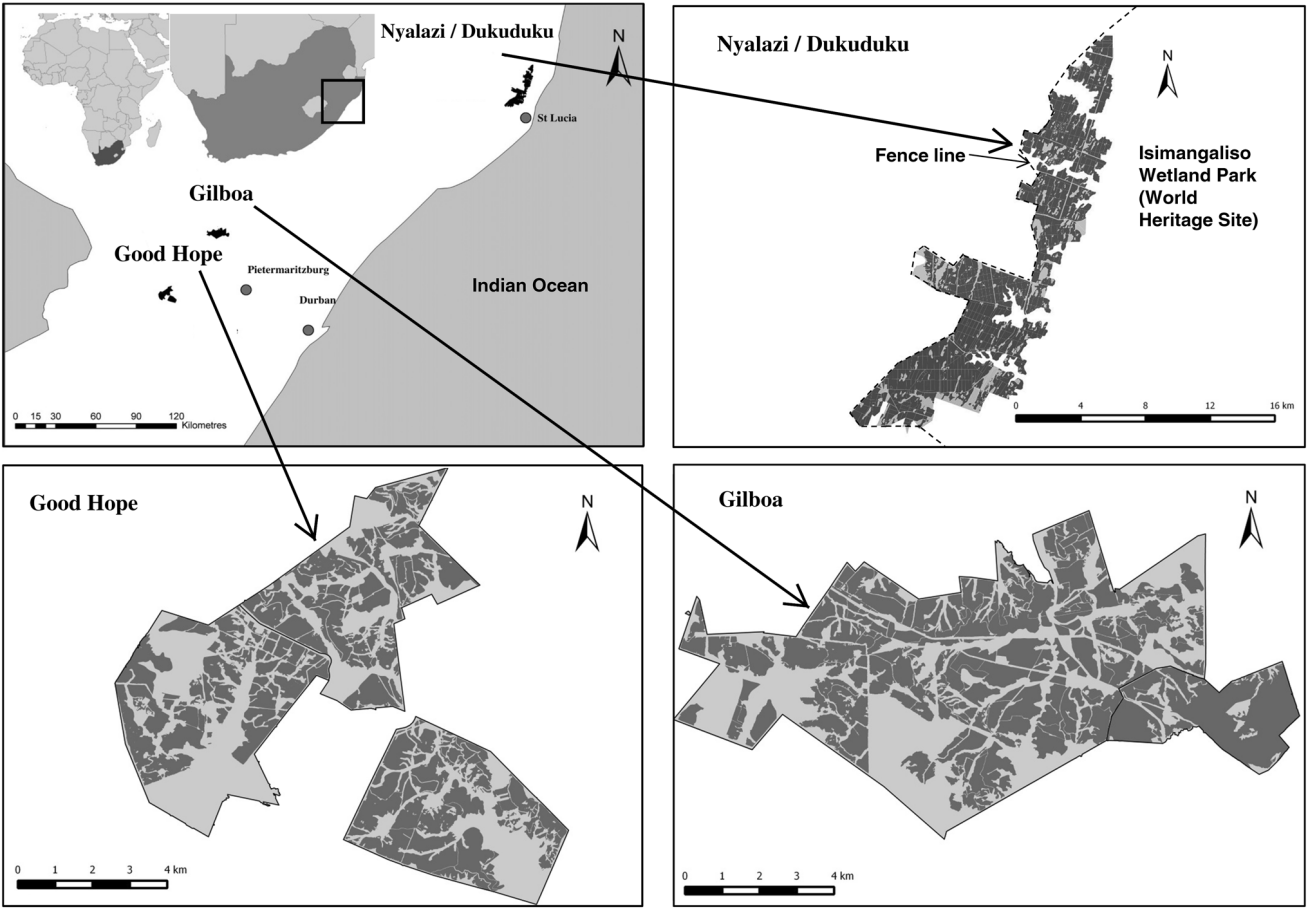
Maps of three plantations with large-scale ecological networks (ENs) in South Africa. The dark areas are timber compartments, while the light areas are the ENs. The plantation/EN in the Nyalazi/Dukuduku is adjacent to a large protected area, with the park now extended as corridors into the plantation area, allowing many large mammals to wander freely among the timber plantation compartments. The matrix surrounding all these plantations/ENs is composed variously of other plantations/ENs, other agriculture, and protected areas

We have adopted retrospective analysis to implement ENs at an extensive spatial scale in the context of plantation forestry in southern Africa (Fig. 1). The South African timber industry has retained >0.5 million ha of remnant habitat in and among exotic tree plantations as ENs. The aim of these ENs is to mitigate biodiversity loss, maintain ecosystem processes and services, and improve the quality of life of local communities, while at the same time ensuring financial viability of the timber matrix. This has been conceptualized into a socio-ecological system and put into a framework for ecosystem provision (Samways et al. 2010). Many of these ENs associated with any one plantation are connected with each other. This extensiveness of ENs is significant as they are subject to the globally significant El Niño-Southern Oscillation (ENSO) (Cai et al. 2014) which arguably is important for how these ENs might be effective given the inevitability of global climate change (Aichi Target 15) (Bakker et al. 2015).

Invoking retrospective analysis is akin to the Precautionary Principle, the principle that we must be sensitive to the complexity and levels of current biodiversity, yet also be cautious because we do not know the extent to which using it will adversely affect those natural resources (Fauna and Flora International 2006). By taking this precautionary approach, there is good reason to put ENs in place as a multipronged approach aimed at ensuring the future of the local biota, their interactions, and services. This is the ‘solution’ that we aim to achieve by retrospective analysis.

**Fig. 2:**
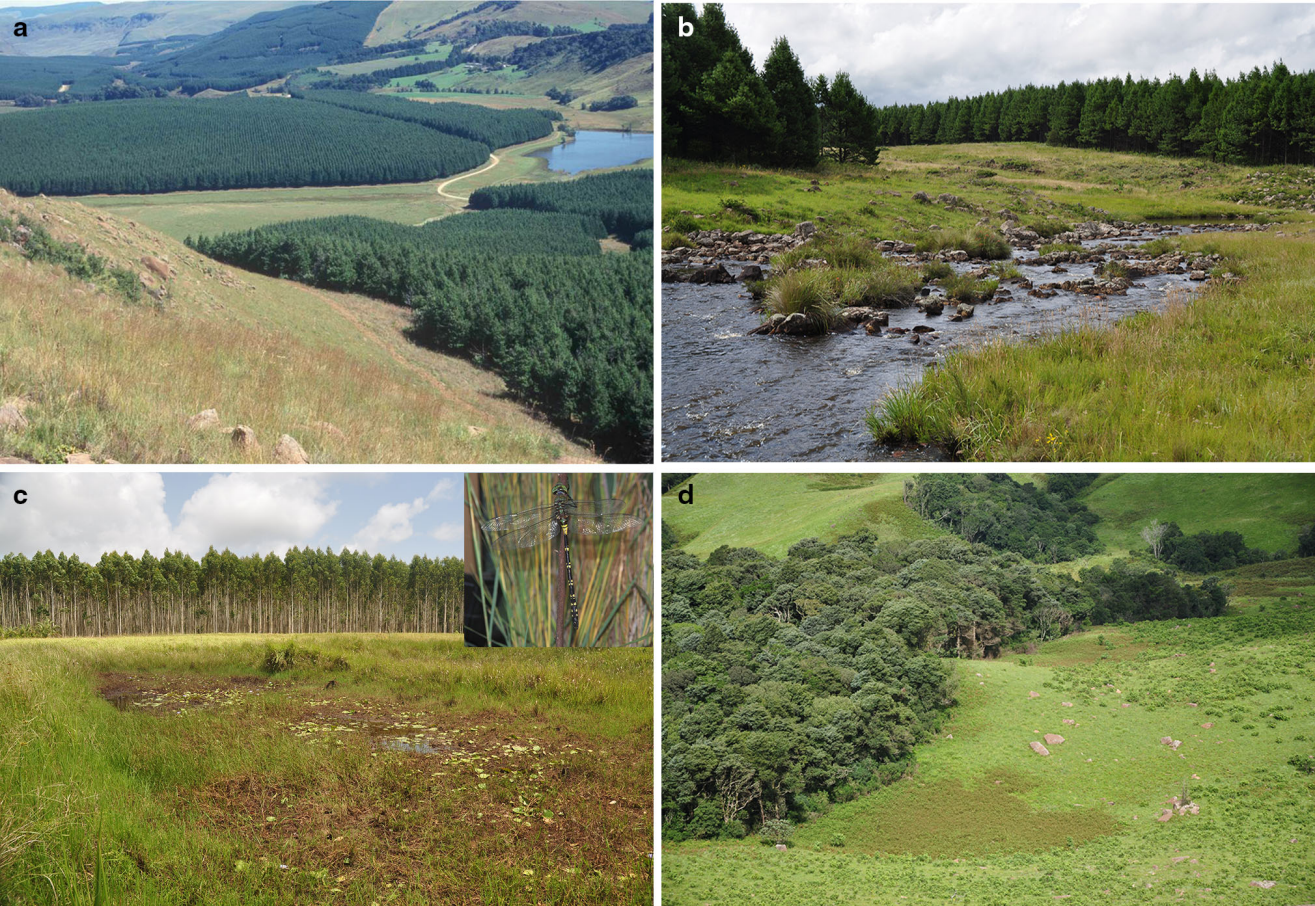
**a** An ecological network (EN) with typical wide and narrow conservation corridors, as well as varied topography. In the far upper left is a protected area adjacent to the EN. **b** These ENs must enable the local ecosystem processes to continue, and this is particularly important for hydrological processes. **c** The operational scale of the mesofilter (features of the landscape) is a major component of these ENs, with rocks, bare ground, and pools (seen here) being conserved alongside vegetation heterogeneity. This particular pool is home to one of the world’s largest dragonflies, the black emperor *Anax tristis* (*inset*). **d** These ENs aim to maintain as much natural heterogeneity as possible. While principally conserving grassland, they importantly also conserve the natural forest patches associated with the variable topography

There has been criticism that plantation forestry using alien trees is harmful to local biodiversity (Neke and Du Plessis 2004), but this view needs unpacking relative to spatial scale. While local biodiversity is changed and impoverished at the small spatial scale of the plantation patch (i.e., timber compartment) Samways and Moore 1991 (re *Pinus* spp.); Samways et al. 1996 (re *Pinus* spp. and *Eucalyptus* spp.); Pryke and Samways 2012b (re *Pinus* spp.), the important point is to focus on the larger spatial scale of the extensive landscape and sub-region. It is at this scale that ENs come into their own as a highly significant conservation measure in and among a production mosaic. These ENs not only provide structural and functional connectivity but are also sufficiently extensive to provide enough remnant habitat so that all local biodiversity and ecosystem processes might be maintained (Samways 2007). Importantly, these ENs are mostly good quality remnant natural grassland and forest, at least in the case of newly established plantations (Fig. 2a). This means that there is equivalent of natural resilience in the system at the landscape and sub-regional scales, especially when ENs are joined together and adjacent to PAs.

We aim to show here that ENs can work in practice, and significantly so for irreplaceable and threatened biodiversity and ecosystem processes in a biodiversity hotspot [the Maputaland-Pondoland-Albany Hotspot (Mittermeier et al. 2005)], and in doing this we address Boitani et al.’s (2007) call for evidence of EN success.

## Freshwater conservation in ecological networks

### River and riparian zones

Rivers are the most threatened of all ecosystems, with declines in biodiversity estimated to be up to five times greater in some rivers than in most degraded terrestrial ecosystems (Dudgeon et al. 2006). This means that the rivers and riparian zones in ENs have to receive immediate attention.

The former approach to regional plantation forestry was to maximize the number of planted trees across the landscape, leading to intensive ‘wall-to-wall’ forestry which was having a major detrimental effect on local biota and on natural processes such as hydrological cycles (Neke and du Plessis 2004). As this former approach had no regard for topography, planting even took place across rain catchment and upper flow areas, often resulting in the cessation of flow of headwater streams and loss of local biodiversity (Lawes et al. 1999). It also led to loss of local livelihoods, as little natural land was left for local human community activities such as honey gathering, planting of crops and, in particular, the grazing of livestock.

There was an allied and additional challenge of an increasingly serious and damaging invasive alien plant problem which has a dire effect upon hydrological processes (Le Maitre et al. 1996, 2004). This issue was addressed by a massive co-operative action with the national Working for Water Programme, the prime aim of which is to remove riparian alien plants, especially trees, to restore water supplies and to engage a large labor force to physically address the problem and give local communities employment (Gorgens and van Wilgen 2004).

The extensive-intensive plantation approach had to change, and could indeed be revolutionized through major new approaches. The already planted landscape could be modified in a way that some of the planted trees are removed to create areas which would be restored, and no longer ever be planted with alien plantation trees. The collectively decided approach, after much discussion among many stakeholders, was to restore hydrological processes by focusing on the position of certain trees that were the prime adverse drivers of hydrological dysfunction. This involved a process of delineation, where afforested land was surveyed for soil types, and plantation trees on hydromorphic soils were removed. This went hand-in-hand with an understanding of the topography, and an initial intuitive suggestion being that the non-afforested areas had to be large enough for effective biodiversity conservation and the maintenance of ecosystem processes. Additionally, invasive alien plants, especially trees in river courses, had to be controlled to restore historic hydrological processes (Le Maitre et al. 1996). Also, restoration of freshwater required the use of effective bioindicators, with dragonflies being used as they are sensitive, taxonomically well-known, and species-level bioindicators (as opposed to use of aquatic macroinvertebrate higher taxa) (Clausnitzer et al. 2012).

Alien plantation trees impoverish the dragonfly fauna <30 m from the water’s edge, but this is the same effect as from indigenous trees (Kinvig and Samways 2000). The critical point is that invasive alien trees, especially *Acacia mearnsii*, which is also a plantation tree, are largely within this 30 m riparian zone. Using both field observation (Smith et al. 2007) and field manipulation experiments (Remsburg et al. 2008), the critical factor adversely affecting the essentially sun-favoring odonate assemblage is the shading effect of plantation trees (Smith et al. 2007). This means that it is essential to keep plantation trees >30 m from the river edge (Fig. 2b).

The challenge of invasive alien trees adversely affecting riparian zones was then addressed by large-scale removal of alien trees which leads to recovery of both abundance and species richness of generalist dragonfly species as soon as the alien trees are removed (Samways and Sharratt 2010). Even specialist endemic species show remarkable resilience by returning 2 years after alien tree removal, with important indicator species for river recovery being identified. This pattern of recovery after alien removal was repeated elsewhere in the region, with dragonfly abundance increasing six-fold and species richness increasing three-fold once the alien trees are removed (Samways and Grant 2006).

Also, riparian invasive alien trees support lower diversity scores for macroinvertebrates than does natural riparian vegetation (Magoba and Samways 2010). Nevertheless, some taxa survive well under the alien trees (e.g., whirligig beetles Gyrinidae) and others increase in abundance (e.g., the fly families Dixidae and Simuliidae). However, indigenous macroinvertebrate diversity improves significantly and begins to return to its historic state within 2 years after alien tree removal, but takes 5 years to fully recover. This recovery is strongly related to recovery of indigenous bushy vegetation, (which for example acts as oviposition sites for endemic damselflies) as well as certain endemic plant bioindicators (e.g., *Prionium serratum*) (Samways et al. 2011).

What this strategic research established was that ENs must include a remnant or restored riparian conservation zone of >30 m either side of the river, and there must be removal of subsequent alien tree regrowth after removal of the mother trees. This has largely been done, with EN riparian zones now mostly free of alien trees and in an ecological condition where future alien tree invasion is much reduced.

In short, success of ENs in terms of recovery and maintenance of the endemic water fauna and flora to a historically functioning ecosystem takes about 5 years, with the dragonfly fauna being identified as excellent bioindicators and umbrellas for freshwater and riparian diversity in general (Smith et al. 2007; Magoba and Samways 2010). This required development of a validation method for river condition in ENs, the Dragonfly Biotic Index (Simaika and Samways 2009), and for comparing rivers within the region and across regions (Simaika and Samways 2012). This validation approach has now been developed into a manual for practitioners charged with maintaining high habitat quality of water courses flowing along the ENs as well as elsewhere (Samways and Simaika 2015).

### Standing water bodies

Firstly, it is essential to conceptualize the difference between flowing and standing waters as the difference has important implications for EN development. Streams and rivers are continuous longitudinal ecosystems which arise in or flow through ENs as conceptualized in the river continuum concept (Vannote et al. 1980). Part of EN development involves maintaining the principal water courses unimpeded by impoundments and embedded within *a* > 60 m EN conservation corridor (Kinvig and Samways 2000; Kietzka et al. 2015). This ensures flowing aquatic corridors that continue to function as they did prior to anthropogenic impact, and also into the future, as envisaged by Auffret et al. (2015).

Lentic systems, in contrast to continuously moving linear lotic ecosystems, although often connected to flowing water, can be viewed principally as patches. This means determining the relative contribution of anthropogenic disturbances and important environmental variables. Having established the value of dragonflies as sensitive and workable bioindicators, this taxon was used for this purpose. Counter-intuitively, natural environmental variables, especially habitat heterogeneity, were found to be more important than the anthropogenic variables in determining the dragonfly assemblages, leading to the conclusion that EN design should incorporate as many natural local variables as possible (Kietzka et al. 2015) (Fig. 2c).

Retrospective analysis can be strategically implemented when there is direct comparison between ENs and a PA as reference condition for naturally functioning ecosystems. When this was done for still water bodies, again using dragonfly bioindicators, the still water bodies in the EN and the PA were found to share 74 % of all species, and share an equal number of range-restricted species. As with lotic species, landscape heterogeneity plays a major role, with variables such as water body size, habitat heterogeneity (including that driven by large mammals creating wallows), elevation, and dissolved oxygen being particularly important. Overall, the freshwater systems in the ENs are equivalent to those in PAs, with the ENs functioning like PAs (Pryke et al. 2015). Furthermore, the historic function of ENs can be restored, so long as alien trees are removed from the riparian corridors, that these corridors are >60 m wide, and especially for standing water bodies, when the historic range of environmental variables and habitat heterogeneity is present.

## Terrestrial biodiversity conservation

### Design variables

ENs are put in place as structural entities to support functional connectivity. Taking a retrospective analysis approach requires a strategic solution, and one way to achieve it is by illustrating, as with freshwater, EN equivalence of terrestrial biota to that in PAs. Where there is high equivalence, there is likely to be a good simile of natural function. However, as the ENs are in a biodiversity hotspot, it means that there is very high spatial heterogeneity among terrestrial biotic communities, especially involving rare species (Pryke and Samways 2015). Once the natural reference level of spatial heterogeneity is incorporated into EN design, other design variables, especially corridor width, and the effect of adjacent historic forest patches, as well as management action, can be implemented to maintain the natural level of biodiversity. The spatial heterogeneity is hierarchical with patch scale β1-diversity (species turnover in a patch/landscape element) and landscape scale β2-diversity (assemblage compositional differences between elements and patches) both being fundamental aspects for determining the conservation value of design parameters for the ENs (Pryke et al. 2013) (Fig. 2d).

Following recognition of the importance of heterogeneity, and its dynamics over time, it is possible to determine levels of equivalence which in terms of plant species composition is greater between sites, whether they are in an EN or a PA, than between the overall EN and PA (Joubert and Samways 2014). For dragonflies, which are amphibiotic, equivalence is 74 %, with heterogeneity making up the other 26 % composed of species exclusive to EN or PA in equal proportions (Pryke et al. 2015). Butterfly equivalence is also high, with only three species in the EN and not in the PA, and two in the PA but not in the EN (Pryke and Samways 2003). Interestingly when a large multi-taxa arthropod assemblage is considered, there are no significant differences in species richness, abundance, or assemblage composition, leading to the important conclusion that ENs can function as extensions of PAs (Pryke and Samways 2012a).

ENs are embedded in a matrix which can be highly influential on local biodiversity (Driscoll et al. 2013). For these ENs in a plantation mosaic, the greatest influence is from edge effects caused by the plantation trees. The significance of these edge effects relates in part to functional connectivity. Among the grassland butterflies of the ENs, only two species entered (and then only <20 m) into the surrounding pine compartments, compared with several species entering adjacent natural forest. Even exotic pines as a patch in a natural grassland matrix can strongly affect grasshopper assemblages <30 m into the grassland, with differences according to aspect (Samways and Moore 1991). When a range of arthropod taxa is considered, species responses vary, with greatest distance from the pine edge to interior being 32 m (Pryke and Samways 2012b). Corridors in the ENs could then be considered only to have interiors when >64 m wide, which interestingly, is the case with freshwater lotic corridors.

**Fig. 3:**
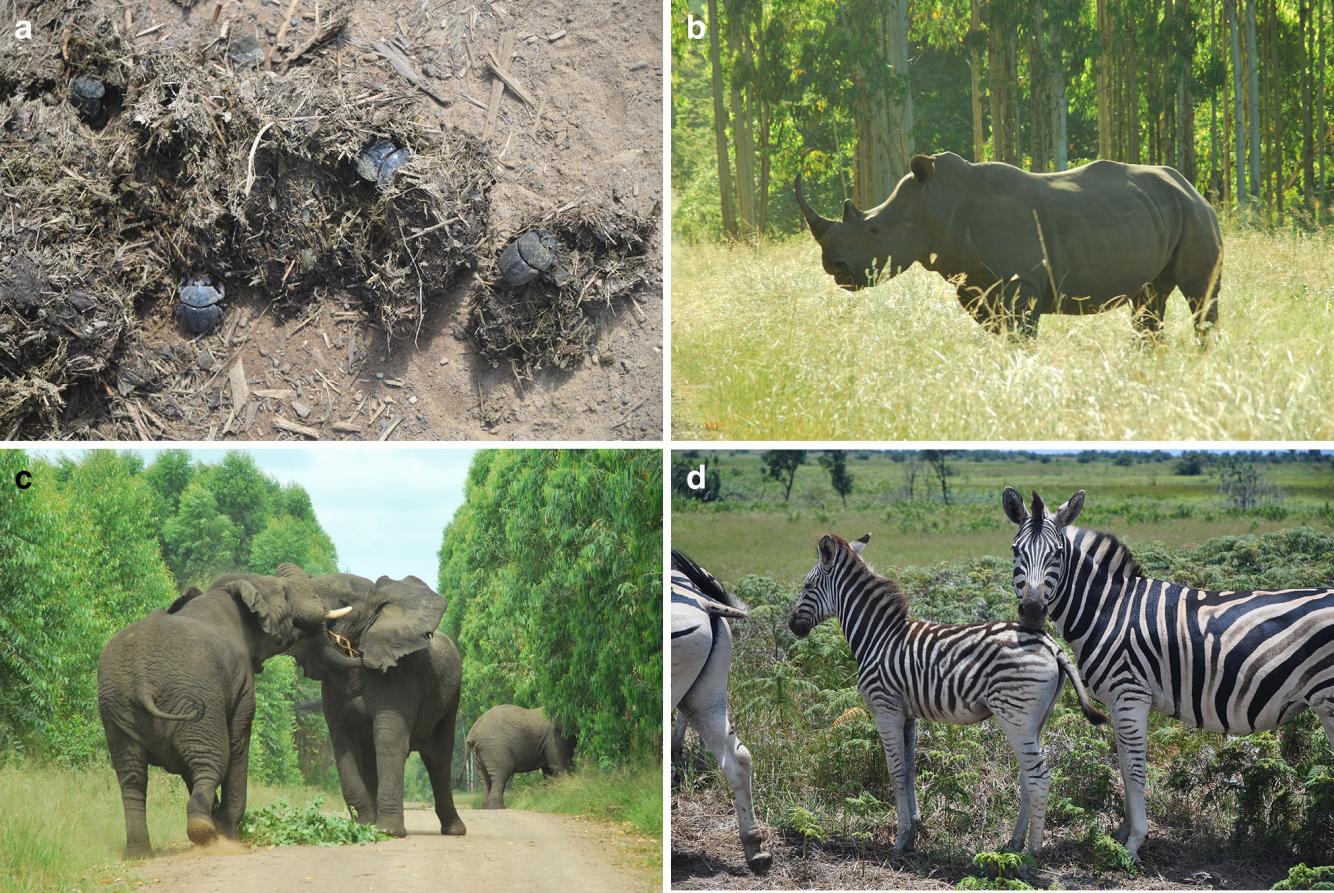
**a** Dung beetles, along with elephants, are conserved in the ecological networks (ENs). **b** A white rhino grazing in an EN. **c** Elephants use the ENs, causing only minimal damage to plantation trees. **d** Megaherbivores are an essential component of the African landscape, and are also a management component of the ENs, keeping them in a historically natural condition

Quality of interiors improves considerably as corridor width in the ENs increases, with those > 250 m wide functioning as natural habitat (Pryke and Samways 2001) and acting to extend the functionality of PAs (Pryke and Samways 2012a). The edge areas are not without biodiversity value, and include edge specialists such as certain ground and dung beetles (Pryke and Samways 2012b), as well as certain grasshopper species (Bazelet and Samways 2011b). Narrow corridors, although not necessarily functioning as historic habitats can nevertheless function as conduits (Hess and Fischer 2001), with butterfly flight speeds being overall 13.3 times greater in narrow than 250-m-wide high-quality corridors (Pryke and Samways 2001). Furthermore, a variety of corridor widths support the greatest range of ant species, with different widths having complementary assemblages. However, it is only the wide corridors which support the full range of dung beetles, a subset of those in wide corridors (van Schalkwyk 2015) (Fig. 3a). Environmental variation, especially elevation and vegetation type are the most important variables for these assemblages, again emphasizing the value of making sure that ENs from a functional point of view maintain historic natural landscape heterogeneity.

As regards birds, specialist species are only present in the large, extensive areas of the ENs where burning regimes are appropriate (Lipsey and Hockey 2010). Where there are no fences between the PA and EN, large mammals freely roam between the two. These include Eland (*Taurotragus oryx*) at higher elevations (±1400 m asl), and African Elephant (*Loxodonta africana*), White Rhinoceros (*Ceratotherium simum*), African Buffalo (*Syncerus caffer*), Giraffe (*Giraffa camelopardalis*), Blue Wildebeest (*Connochaetes taurinus*), Plains Zebra (*Equus burchelli*) and others at lower elevations (±20 m asl), which seek good habitat patches (‘grazing lawns’) (Fig. 3b–d). Some small but rare species such as Oribi (*Ourebia ourebi*) can also benefit from ENs.

The ENs, while mostly indigenous grassland, also contain natural forest patches honed by natural fires over many millennia, with these fires having reduced forested areas into numerous small remnant patches spread throughout the grassland (Eely et al. 1999). These patches support considerable additional species and interactions over the grassland, and can be considered as part of the historic functional landscape (Pryke and Samways 2012b).

### Management variables

Management of ENs plays an important role as emphasized by sensitive grasshoppers showing that management is consistently 2–5 times more influential than design (Bazelet and Samways 2011a). However, it is not that these two factors are mutually exclusive, with firebreaks for example determining point biodiversity both in terms of design and management. Working on plants, Joubert (2014) concluded that past disturbances and current management practices should be taken into account and integrated into the future design of ENs.

**Fig. 4:**
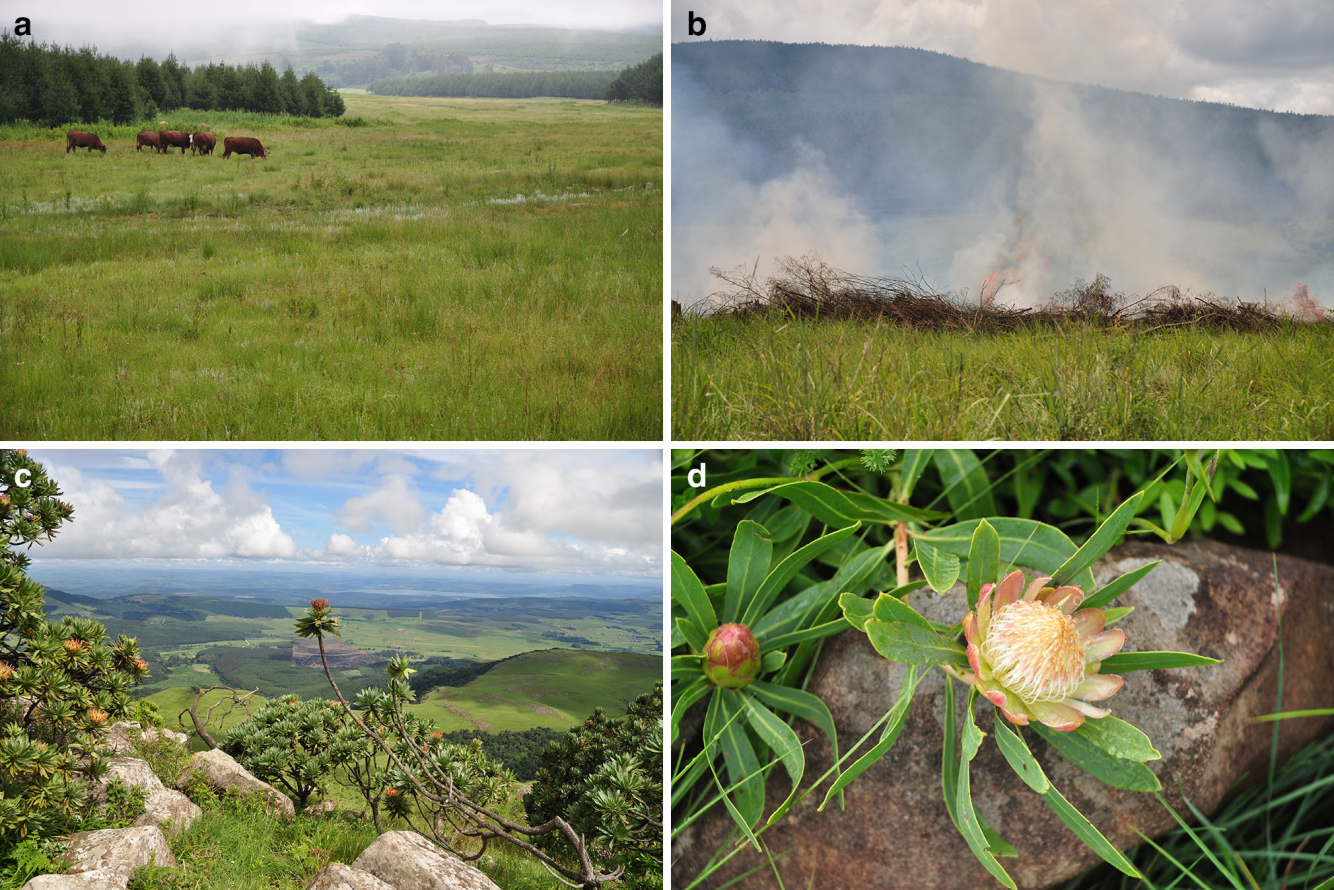
**a** The ENs must provide for the local human communities, and this involves grazing of cattle, among other activities. Where there is less density of megaherbivores than in the past, cattle serve as grazing substitutes which then maintain historic biodiversity. **b** Management of the ENs using artificial fires involves on the one hand, mimicking natural conditions, and on the other, protects the timber crop. **c** A plantation/EN with highly varied topography and one which has very high conservation value (Gilboa). **d** The operational scale of the fine filter (species conservation) overlays the coarse filter (landscape conservation), with rare and endemic plants, fungi`, and animals receiving special conservation status. Shown here is the rare dwarf-grassveld protea (*Protea simplex*)

As well as for biodiversity conservation, ENs have been implemented to enable local people to acquire greater dispensation, especially to graze livestock at sustainable stocking rates (Fig. 4a). Such artificial grazing is not of great concern for the indigenous biodiversity, as cattle at moderate stocking rates are a good surrogate as grazers where there has been loss of the historic large megaherbivores (Samways and Kreutzinger 2001; Joubert 2014). Not that all of the wild game animals have been lost, but their abundance was naturally higher in the past. Today, they are more restricted in their area of occupancy, and with less opportunity to move across the landscape, except in the ENs and PAs with no fences between the two.

Fire is important in two respects (1) to protect the timber compartments from runaway fires (O’Connor et al. 2004), and (2) to mimic the natural fire regime in this naturally fire-driven ecosystem (Uys et al. 2004; Little et al. 2012; Joubert et al. 2014) and for the establishment of firebreaks for protection of the plantation trees from runaway fires, a serious threat to production stands in this geographical area (Fig. 4b).

When fire frequencies are high (e.g., annual burning of firebreaks both in ENs and PAs), there is homogenization of plant assemblages but not a reduction of plant species richness in the burned areas compared to those in wide corridors of the ENs and in the PAs (Joubert et al. 2014). Regularly burned areas also favor a particular grasshopper assemblage characterized by early colonizers (Bazelet and Samways 2011b).

While the ENs are largely free of invasive alien trees, which mostly colonize water courses and have been removed, there is still a localized problem in middle-elevation (1000–1500 m a.s.l.) ENs with alien bramble (*Rubus cuneifolius*) impacting on local pollinator networks (Hansen 2015). However, some alien plants, such as *Verbena bonariensis*, carry an advantage as a nectaring plant for many of the local butterflies, so increasing their local distribution (Pryke and Samways 2003).

In short, corridor width largely equates to increased habitat quality in these remnant ENs. When habitat quality is high (i.e., corridors are >250-m wide and represented by indigenous vegetation). When management is integrated with this quality design, and cattle grazing is moderated (Bullock and Samways 2005; Joubert et al. 2014), alien bramble is removed, and fire frequencies are at the historic rate, the corridors of the ENs all show equal diversity along their lengths [e.g., butterflies (Pryke and Samways 2003), grasshoppers (Bazelet and Samways 2011b), arthropods on flowers (Bullock and Samways 2005), and ground-living arthropods (Pryke and Samways 2012b)] indicating a high level of connectivity. The converse of this is seen when fragments of remnant grassland are not connected by high-quality corridors and are isolated by being surrounded by plantation trees, which causes morphological change (anthropovicariance: Williams 2002) in a grasshopper species in these remnant islands (Bazelet and Samways 2014). The implication is that the EN corridors enable maintenance of the natural genetic flux which is otherwise interrupted by isolation.

## Validation

The iterative process for instigation and improvement of these ENs has been as follows: design → implement design → validate design → redesign → implement redesign → manage → validate management → assess value against reference sites i.e., PAs. To validate this process, ENs have been compared to nearby PAs using sensitive bioindicators at the appropriate spatial scale. The following were finally selected: the primary producers (plants, especially the local assemblages of plants at the spatial scale of a few meters) (Joubert et al. 2014; Joubert and Samways 2014), responsive herbivores (grasshoppers) (Bazelet and Samways 2011b), anthophiles/herbivores (butterflies, which strongly cross-correlated with grasshoppers) (Bazelet and Samways 2012), indicators of mammal residence and habitat heterogeneity (dung beetles) (Pryke et al. 2013) (Fig. 3a), and predators (spiders) (Pryke and Samways 2012a, b) (dragonflies) (Kietzka et al. 2015), among others. However, a multi-taxon approach is essential if the true range of biodiversity as a whole is to be represented (Pryke and Samways 2012c). These various bioindicators enable the iterative process to be followed through, so as to determine the design of the ENs, their optimal management, and their value and resilience in comparison with the adjacent PAs (i.e. natural reference areas).

## Coarse filter, mesofilter, and fine filter

While these ENs were crafted and are being managed as a coarse-scale, landscape activity, there is continual cognizance of the importance of smaller spatial scales. This is particularly so as these ENs are in a biodiversity hotspot (the Maputaland-Pondoland-Albany Hotspot), with a wide taxonomic range of endemic species and interactions. These species are often associated with special habitats, and so there is recognition of the importance of habitat heterogeneity as it relates to the mesofilter (features of the landscape) (Hunter 2005). Indeed, the habitat heterogeneity associated with these ENs is manifested strongly at the mesoscale of tens of meters, with the type of local biotope at this spatial scale (e.g., patterns of rocks in grassland, water pools, damp areas, patches of bare soil, logs) being important for the local dispersion patterns of the biota (Crous et al. 2013) (Fig. 4c), as is the toposcape (Samways 1990) (Fig. 2a). At the still smaller scale of arthropods associated with endemic flowering plants, as long as the individual plant is present, even in a narrow, disturbed corridor, the historic complement of arthropods is present (Bullock and Samways 2005).

When ENs are adjacent to a PA, they are an effective extension of that PA for a whole range of taxa and functional groups. This means that the fauna has the option to use the ENs when conditions are more suitable in them, than in the PA at the time. The extensions can also include habitats for certain rare and threatened species (Fig. 4d) that are not known elsewhere, even in PAs (e.g., the white red-hot poker *Kniphofia leucocephala*, Red Listed as Critically Endangered). ENs can also be for threatened species that require effective management (Lu and Samways 2002) or monitoring for breeding success (e.g., Karkloof blue butterfly *Orachrysops ariadne*, Red Listed as Endangered) (Armstrong and Louw 2013).

## Ecological networks and global climate change

ENs as extensions of PAs are significant as they lie within a region exposed to globally significant ENSO effects (Cai et al. 2014). ENs can increase current (and future—given local and global climate change) effectiveness of PAs in terms of increased space per se and also for providing spatial options when conditions are adverse e.g., very dry or wet. For example, dragonflies move in and out of the local landscape during wet and dry phases (Samways and Niba 2010).

ENs must be sufficiently resilient, permeable, and be able to accommodate global climate change. Organisms in this and other ENSO areas have likely been honed over the millennia to survive the climatic oscillations and, through natural selection, have developed strategies to survive it, either by moving around (both horizontally, and/or vertically over an elevation gradient). As these ENs overall cover a great elevation gradient (0–1800 m a.s.l.), they have an inherent ability to cater for the vagaries of not just ENSO but potentially also global climate change, with species distribution models of dragonflies for the years 2050 and 2080 suggesting no extinctions but considerable species turnover (Simaika and Samways 2015). However, ENSOs are principally about relative amounts of precipitation and not necessarily warming and cooling. Nevertheless, as well as the wet and dry cycles, there are changes in temperature associated with different seasons and years, with the organisms already ‘pre-adapted’ to survive global climate change.

One rationale behind using these ENs as conservation measures for climate change is that whole communities and ecosystem processes are being maintained based on retrospective analysis in the design and placement process. Single species studies provide little insight into how whole communities might be affected under future climate scenarios, with insects and plants for example having been coupled and decoupled with climate changes of the Upper Quaternary (Ponel et al. 2003). Additionally, ENs must accommodate increased flooding and drought as well as temperature changes (Pearce 2006; Zalasiewicz and Williams 2012). The likely temperature increases are largely catered for, as the ENs cover a range of elevations, with differentials of 200 m a.s.l. over any one landscape being common (Figs. 2a, d, 4c). This equates to tolerating an overall 1.2 °C change in temperature.

## Conclusions

The challenge put out by Boitani et al. (2007) that there is still little evidence to show that ENs can conserve biodiversity in the long term and that ecosystem function will be maintained is an appropriate one. However, to address such a challenge requires a huge amount of research, making it essential that a strategic retrospective analysis approach is taken to hone data for an effective solution. We have shown here that ENs can indeed conserve biodiversity, although long-term issues are difficult to address, and as we show here are effectively the same as asking whether PAs can do the same.

So what have we learnt from this retrospective analysis of ENs in South Africa and what is applicable globally? Firstly, there must be a good understanding of the level of heterogeneity across the landscape, and at various spatial scales from that of the mesofilter, where features are critical to many taxa and interactions, through the landscape to the sub-regional level for the maintenance of overall hydrological processes among other processes. Furthermore, the word ‘function’ is fraught with further challenges, and a way forward is to use substantial and historically sound PAs as reference, on the assumption that if the ENs are equivalent to the PA in terms of their species composition and interactions, there is likely to be good simulation of the naturally historic fully ‘functional’ ecosystems. This determination of equivalence between ENs and PAs is a benchmark of EN condition.

All landscapes are dynamic and change over time, making it essential that ENs not only accommodate variations of weather and climate, but also be able to buffer future anthropogenically induced climate change. This we can never actually know until it happens, not just with ENs but with ecosystems in general.

There are other demands upon ENs in terms of delivering success. Firstly, there must be management of the landscape both to mimic natural disturbance such as fire regimes and grazing. Firebreaks must be implemented to protect the production timber, but these areas are not without biodiversity value and by supporting early succession species are complementary to the wide corridors which simulate PAs. Furthermore, there is the socio-ecological component, where local communities must also be accommodated, and by introducing cattle into the ENs there is the double benefit of providing livelihoods and providing the intermediate disturbance which is the historic condition when large herbivores were more extensive across the landscape. Overall, these ENs provide a win–win situation where conservation and agroforestry production can operate in the same overall space for the benefit of both.
